# Dynamic look-ahead feedrate scheduling method based on sliding mode velocity control

**DOI:** 10.1038/s41598-024-66203-w

**Published:** 2024-07-04

**Authors:** Liuquan Wang, Qiang Liu, Pengpeng Sun, Shisheng Lv, Ruijie Yang, Zhiqi Yang

**Affiliations:** 1 School of Mechanical Engineering and Automation, Beihang University, Beijing, 100191 China; 2 Jiangxi Research Institute of Beihang University, Nanchang, 330096 China; 3 Mechanics and Acoustics Division, National Institute of Metrology, Beijing, 100029 China; 4 Beijing Engineering Technological Research Center of High-Efficient and Green, CNC Machining Process and Equipment, Beijing, 100191 China; 5 Research and Application Center of Advanced, CNC Machining Technology, State Administration of Science, Technology and Industry for National Defense, Beijing, 100191 China

**Keywords:** Feedrate scheduling, Dynamic look-ahead, Sliding mode control, Tracking error, Mechanical engineering, Engineering

## Abstract

In the feedrate scheduling of complex curve direct interpolation, dynamic constraints such as axis acceleration and jerk are related to the actual state of the tool. Most existing methods convert dynamic constraints to velocity constraints at sampling points. However, it cannot guarantee the dynamic constraints are satisfied between sampling points. Addressing the issue, this paper proposes a dynamic look-ahead feedrate scheduling method based on sliding mode velocity control, which generates the motion command considering dynamic constraints in every interpolation cycle. To dynamically generate commands based on the current tool state, the acceleration and deceleration method based on sliding mode velocity control has been proposed, which can control tool state to transition to the command state with any initial state. To ensure sufficient distance for acceleration and deceleration, this paper uses braking distance to dynamically estimate the look-ahead distance. Then the minimum value within the look-ahead interval is selected as the command velocity for this scheduling cycle and the actual motion command is determined based on the dynamic constraints of each axis. Simulation and experiment results prove that compared with the existing method, this method effectively reduces the overshoot of dynamic constraints without significantly increasing the machining time. The analysis of real-time computation time has demonstrated the potential of the method proposed in this paper for real-time applications.

## Introduction

As the standard form for curves in the STEP standard, Non-Uniform Rational B-Splines (NURBS) curves are widely used for the geometric expression of complex parts. Direct interpolation for NURBS curves could avoid discretizing the continuous NURBS curve to straight lines and arcs and improve machining efficiency and precision. In machining process, high-accuracy surface quality requires that tool motion satisfy constraints such as chord error, tangential acceleration, and tangential jerk^1^. Additionally, surface quality is also affected by servo axis motion performance such as servo tracking bandwidth, maximum acceleration and jerk^2^. Feedrate scheduling is an important component of NURBS direct interpolation, which can fulfill the requirement of machining accuracy and quality by adjusting the feedrate to satisfy constraints in geometry, kinematics and dynamics^3^. The common feedrate scheduling methods for NURBS direct interpolation can be divided into acceleration and deceleration(A/D) method and time-optimal optimization (TOO) method^4^.

The TOO method converts the feedrate scheduling to an optimal control problem and usually optimizes the velocity at sampling points to achieve maximum machining efficiency. After obtaining the time-optimized velocity at discrete sampling points, a continuous *u-v* curve can be fitted by spline to obtain the velocity at any position. Liu et al.^5^ used the square of the velocity as optimization variable, and considered the kinematics and dynamics constraints of the machine tool. Lu et al.^6^ employed a predictive deceleration method to predict switching points of jerk to ensure jerk constraints. Zhang et al.^4^ and Xiao et al.^7^ converted tracking error constraints into linear combinations of tangential velocity, acceleration and jerk, and have taken them into consideration in optimal control. Chen et al.^8^ linearized the contour error as a function of velocity and used the Frenet framework to determine the accurate upper limit of velocity under the contour error constraint. To avoid velocity fluctuation caused by spline fitting, Zhao et al.^9^ adopted A/D method to scheduling the feedrate between sampling points after obtaining time-optimal velocity using optimal control. Yang et al.^10^ used particle swarm optimization to optimize the control nodes of the fitted *u-v* curve. Although TOO can achieve maximum machining efficiency, the computational load limits the use of TOO in real-time environments, for it hard to respond to overridechanges.

The A/D method generally divides the whole curve into sub-curve segments according to the velocity at sampling points. And for each sub-curve, a fixed A/D model is employed to generate the velocity profile in this sub-curve. The most common A/D model is S-shaped model^11^ and here are 17 S-shaped velocity curve profiles according to different conditions^12^. Traditional S-shaped A/D method causes jerk impact. To reduce the jerk impact during the manufacturing process, some jerk planning methods based on trigonometric function^13,14^, sigmoid function^15^ and quartic function^16^ were proposed. However, these approaches increase the complexity of curve classification discussions. Wu et al.^17^ proposed a scheduling method combining quartic S-shaped and cubic S-shaped where quartic S-shaped is used to avoid jerk impact and cubic S-shaped is used to ensure machining efficiency. Ren et al.^16^ proposed a method of segment merging to reduce A/D stages. Sang et al.^18^ used morphological filters to optimize the feedrate limitation profile . Jia et al.^19^ proposed the concept of the velocity sensitive region, in which a uniform rate scheduling was applied to avoid exceeding constraints. Sun et al.^20^ schedule the period of velocity stage to be an integer multiple of servo control cycle to eliminate velocity fluctuations. Currently, intelligent control algorithms in CNC systems, such as adaptive cutting force control^21^, often require changing override real-time. To meet the requirements, many scholars have developed dynamic look-ahead feedrate scheduling methods based on A/D methods. Sun et al.^22^ proposed an acceleration look-ahead method with sin^2^ acceleration curve to avoid frequent acceleration fluctuations during short segments. Zhang et al.^23^ proposed an iterative method that detects exceeding constraint points by pre-interpolation and re-divides sub-segments at exceeding constraints points. However, the iteration time is uncontrollable. Song et al.^24^ proposed a dynamic moving look-ahead window method which uses braking distance to estimate look-ahead window length. Sun^25^ proposed a dynamic look-ahead method, which constructs a hyperbolic tangent function relationship between velocity and acceleration to generate commands based on tool state. However, this method cannot guarantee stable arrival at end point of curve and its stability has not been proven.

As the complexity of parts increases, in the finishing stage, machining stability is more important than efficiency, and it is required to satisfy constraints such as chord error, axis velocity, axis acceleration and axis jerk throughout the entire machining process. Only related to the geometric properties of the curve, static constraints such as chord error and axis velocity can be converted to tangential velocity constraints. However, dynamic constraints such as axis acceleration and jerk are related to the actual state of the tool. Existing methods often assume that the tool’s acceleration and jerk are zero, thereby converting dynamic constraints to velocity constraints at sampling points. However, it cannot guarantee satisfying constraints between sampling points.

To address the needs for real-time override adjustment and dynamic constraints, this paper proposes a dynamic lookahead method based on sliding mode velocity control (SMVC). Compared to S-shaped A/D which constructs a relationship between jerk and time, SMVC generates commands using current tool state, which can adjust jerk more flexibly. Based on SMVC, the dynamic look-ahead feedrate scheduling considers dynamic constraints in every interpolation cycle, achieving consideration of dynamic constraints in the entire machining process. The remainder of this manuscript is organized as follows. “Principle of proposed method” details the principles of proposed scheduling method. “Sliding mode velocity control acceleration and deceleration method” introduces the sliding mode velocity control method. “Feedrate scheduling method” introduces the specific implementation of proposed scheduling method. And “Simulation” and “Experiment” present the simulation and experimental results.

### Principle of proposed method

**Figure 1:**
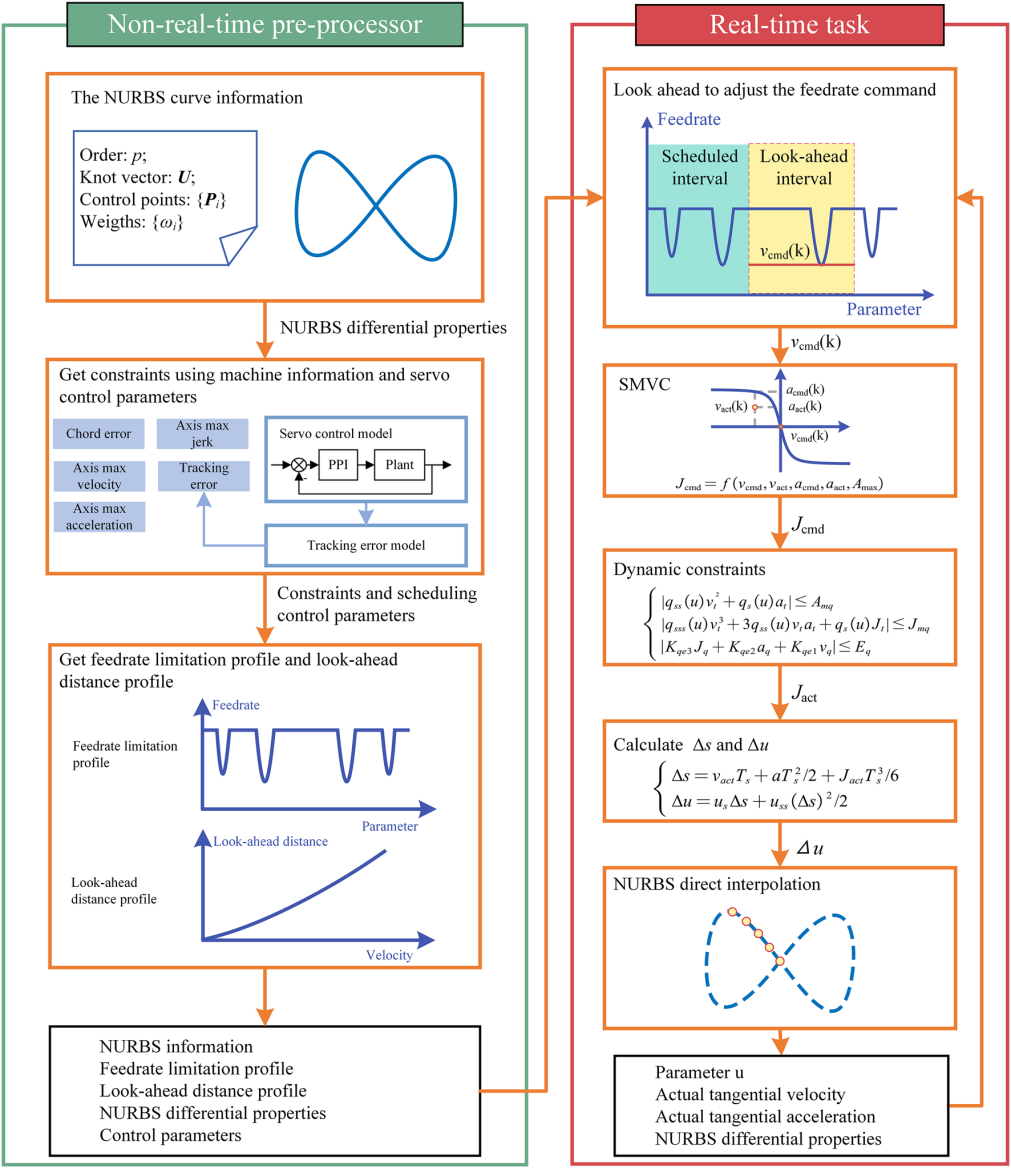
Schematic diagram of dynamic look-ahead feedrate scheduling method based on SMVC.

The dynamic look-ahead feedrate scheduling method is shown in Fig. 1. The feedrate scheduling method is divided into two stages: non-real-time pre-processing and real-time tasks. The non-real-time pre-processing stage realizes the sampling of NURBS to obtain the differential properties of NURBS at sampling points. Combined with the feedrate constraints model such as chord error and axial velocity, the feedrate limitation profile satisfying the constraints is obtained. Meanwhile, the relationship between feedrate and look-ahead distance is established. The above information will be saved in the buffer to support the real-time tasks.

In the real-time task stage, the look-ahead distance of current period is calculated firstly according to the actual feedrate and the look-ahead distance function. After seeking the look-ahead interval, the minimum value of feedrate limitation profile in this interval is to serve as the command feedrate ${v}_{\text{cmd}}\left(k\right)$ of this period. Then SMVC method calculates the desired tangential acceleration and jerk command ${a}_{\text{cmd}}\left(k\right), {J}_{\text{cmd}}(k)$ using the current tool status. Subsequently, dynamic constraints are used to adjust the ${a}_{\text{cmd}}\left(k\right)$ and ${J}_{\text{cmd}}(k)$ and the actual jerk command ${J}_{\text{act}}(k)$ satisfying dynamic constraints is derived. If there is no solution that meets the dynamic constraints, then ${J}_{act}\left(k\right)$ only needs to meet the maximum tangential jerk constraint. Once ${J}_{\text{act}}(k)$ is obtained, the arc length increment $\Delta s(k)$ and parameter increment $\Delta u(k)$ can be calculated. According to the $\Delta u(k)$, the command position and NURBS differential properties of next period can be obtained.

### Sliding mode velocity control acceleration and deceleration method

**Figure 2:**
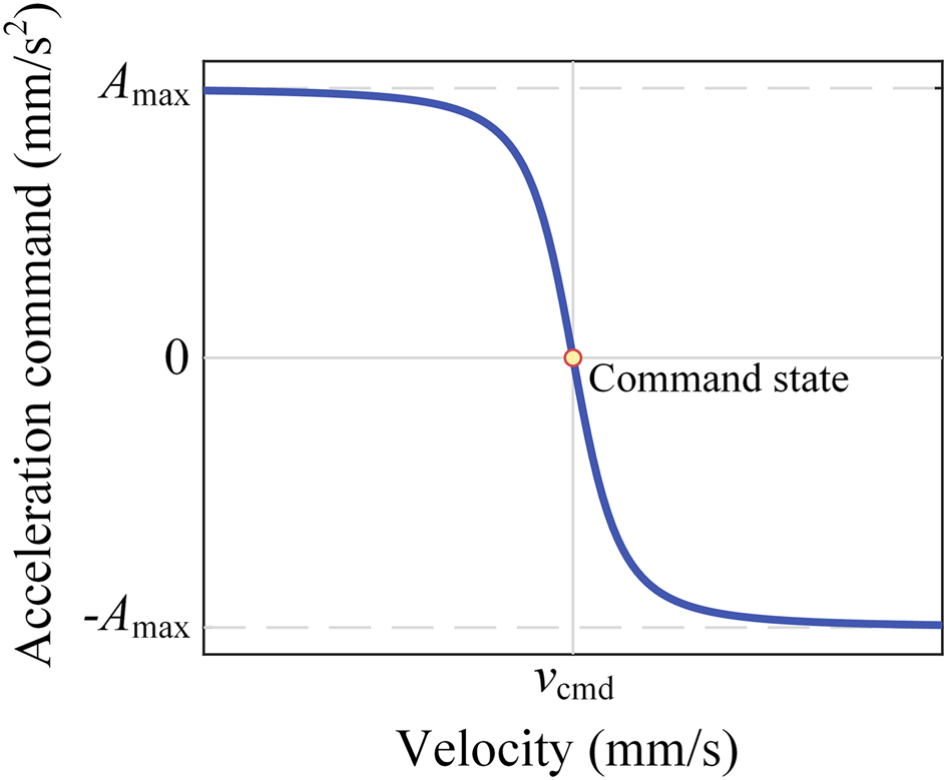
Graph of Eq. (1) on phase plane.

Define tool tip state $${\varvec{x}}={\left[\begin{array}{cc}{v}_{\text{act}}& {a}_{\text{act}}\end{array}\right]}^{T}$$, where ${v}_{\text{act}}$ represents the tool tip’s velocity and ${a}_{\text{act}}$ represents the tool tip’s acceleration. The command state can be represented as $${{\varvec{x}}}_{\text{cmd}}={\left[\begin{array}{cc}{v}_{\text{cmd}}& 0\end{array}\right]}^{T}$$,where the ${v}_{\text{cmd}}$ represents the command tool tip’s velocity. The A/D process is the process that tool tip’s state transitions to command state ${{\varvec{x}}}_{\text{cmd}}$ from current state ${\varvec{x}}$. And in this process, the velocity and acceleration should be continuous and ${a}_{\text{act}}\in \left[-{A}_{\text{max}},{A}_{\text{max}}\right]$. Consider the following relationship between ${a}_{\text{cmd}}$ and ${v}_{\text{act}}$

1 $$\begin{array}{c}{a}_{\text{cmd}}={A}_{\text{max}}\frac{{K}_{1}\left({v}_{\text{cmd}}-{v}_{\text{act}}\right)}{\sqrt{1+{\left({K}_{1}\left({v}_{\text{cmd}}-{v}_{\text{act}}\right)\right)}^{2}}}\end{array}$$

where ${K}_{1}$ is a positive coefficient. The graph of Eq. (1) on phase plane is shown in Fig. 2.

If ${a}_{\text{cmd}}$ satisfies Eq. (1), it will be continuous and in $\left[-{A}_{\text{max}},{A}_{\text{max}}\right]$. To prove the stability of control method described by Eq. (1), let ${v}_{\text{e}}={v}_{\text{cmd}}-{v}_{\text{act}}$ and ${a}_{e}$ be the first-order derivative of the ${v}_{e}$ with respect to time $t$, which can shift command status from $${\left[\begin{array}{cc}{v}_{\text{cmd}}& 0\end{array}\right]}^{T}$$ to $${\left[\begin{array}{cc}0& 0\end{array}\right]}^{T}$$. Equation (1) becomes

2 $$\begin{array}{c}{a}_{\text{e}}={\dot{v}}_{\text{e}}=-{A}_{\text{max}}\frac{{K}_{1}{v}_{\text{e}}}{\sqrt{1+{\left({K}_{1}{v}_{\text{e}}\right)}^{2}}}\end{array}$$

If ${\left[{v}_{\text{e}},{a}_{\text{e}}\right]}^{T}$ can converge to ${\left[\text{0,0}\right]}^{T}$, it implies that ${\left[{v}_{\text{act}},{a}_{\text{act}}\right]}^{T}$ can converge to $${\left[\begin{array}{cc}{v}_{\text{cmd}}& 0\end{array}\right]}^{T}$$.

Define Lyapunov function

3 $$\begin{array}{c}{V}_{1}=\frac{1}{2}{v}_{\text{e}}^{2}\end{array}$$

${V}_{1}$ is obviously positive definite and

4 $$\begin{array}{c}{\dot{V}}_{1}={v}_{\text{e}}{\dot{v}}_{\text{e}}=-{A}_{\text{max}}\frac{{K}_{1}{v}_{\text{e}}^{2}}{\sqrt{1+{\left({K}_{1}{v}_{\text{e}}\right)}^{2}}}\end{array}$$

When ${v}_{\text{e}}\ne 0$, ${\dot{V}}_{1}\ne 0$. It means that if tool tip system follows Eq. (1), it is asymptotically stable near $${\left[\begin{array}{cc}{v}_{\text{cmd}}& 0\end{array}\right]}^{T}$$.

Due to the constraint on jerk, it is necessary to ensure the jerk of Eq. (1) satisfying the limit. There is

5 $$\begin{array}{c}{J}_{\text{cmd}}=\frac{\text{d}{a}_{\text{cmd}}}{\text{d}t}=\frac{\text{d}{a}_{\text{cmd}}}{\text{d}{v}_{\text{act}}}\frac{\text{d}{v}_{\text{act}}}{\text{d}t}=\frac{\text{d}{a}_{\text{cmd}}}{\text{d}{v}_{\text{act}}}{a}_{cmd}=-\frac{{A}_{\text{max}}^{2}{K}_{1}^{2}\left({v}_{\text{cmd}}-{v}_{\text{act}}\right)}{{\left(1+{K}_{1}^{2}{\left({v}_{\text{cmd}}-{v}_{\text{act}}\right)}^{2}\right)}^{2}}\end{array}$$

Furthermore,

6 $$\begin{array}{c}\frac{\text{d}{J}_{\text{cmd}}}{\text{d}{v}_{\text{act}}}=-{A}_{\text{max}}^{2}{K}_{1}^{2}\frac{1-3{K}_{1}^{2}{\left({v}_{\text{cmd}}-{v}_{\text{act}}\right)}^{2}}{(1+{K}_{1}^{2}{\left({v}_{\text{cmd}}-{v}_{\text{act}}\right)}^{2}{)}^{3}}\end{array}$$

Letting $\frac{\text{d}{J}_{\text{cmd}}}{\text{d}{v}_{\text{act}}}=0$, there are ${v}_{1}={v}_{\text{cmd}}-\frac{\sqrt{3}}{3{K}_{1}},{v}_{2}={v}_{cmd}+\frac{\sqrt{3}}{3{K}_{1}}$. In the interval $(-\infty ,+\infty )$, it can be observed that

7 $$\begin{array}{c}\begin{array}{c}\text{max}\left\{{J}_{\text{cmd}}\right\}=\text{max}\left\{J\left(+\infty \right),J\left({v}_{1}\right)\right\}=\frac{3\sqrt{3}}{16}{A}_{\text{max}}^{2}{K}_{1}\\ \text{min}\left\{{J}_{\text{cmd}}\right\}=\text{min}\left\{J\left(-\infty \right),J\left({v}_{2}\right)\right\}=-\frac{3\sqrt{3}}{16}{A}_{\text{max}}^{2}{K}_{1}\end{array}\end{array}$$

Given the maximum acceleration as ${J}_{\text{max}}$, the ${K}_{1}$ should satisfy

8 $$\begin{array}{c}{K}_{1}\le \frac{16}{3\sqrt{3}}\frac{{J}_{\text{max}}}{{A}_{\text{max}}^{2}}\end{array}$$

When the tool tip state satisfies Eq. (1) and ${K}_{1}$ satisfies Eq. (8), the system will converge to the command state ${{\varvec{x}}}_{\text{cmd}}$**.** For states not satisfying Eq. (1), a sliding mode control method can be designed to make them approach the ideal state first.

Define sliding mode surface as

9 $$\begin{array}{c}{s}_{\text{c}}={A}_{\text{max}}\frac{{K}_{1}\left({v}_{\text{cmd}}-{v}_{\text{act}}\right)}{\sqrt{1+{\left({K}_{1}\left({v}_{\text{cmd}}-{v}_{\text{act}}\right)\right)}^{2}}}-{a}_{\text{act}}\end{array}$$

When ${s}_{\text{c}}$ = 0, the current tool state satisfies Eq. (1). When ${s}_{c}\ne 0$, it implies that the tool state is not on the sliding mode surface. By controlling ${s}_{\text{c}}$ to tend towards 0, the tool state can be ensured to approach the sliding mode surface. Consider following equation:

10 $$\begin{array}{c}{\dot{s}}_{\text{c}}=-\frac{{A}_{\text{max}}{K}_{1}{a}_{\text{act}}}{{\left(1+{K}_{1}^{2}{\left({v}_{\text{cmd}}-{v}_{\text{act}}\right)}^{2}\right)}^\frac{3}{2}}-{J}_{\text{act}}=-{K}_{2}{s}_{\text{c}}\end{array}$$

where ${J}_{\text{act}}$ is the jerk of tool tip and ${K}_{2}$ is a positive coefficient. For the system described by Eq. (10), the Lyapunov function ${V}_{2} = {s}_{\text{c}}^{2} / 2$ is chosen. It can easily be proven that ${V}_{2}$ is positive definite and ${\dot{V}}_{2}$ is negative definite. It implies ${s}_{\text{c}}$ can stably converge to 0.

Increasing ${K}_{2}$ can improve the system's performance, but if ${K}_{2}$ is too large, it may cause chatter on the sliding mode surface. In this paper, ${K}_{2}$ is taken as $1/{T}_{\text{s}}$, where ${T}_{\text{s}}$ is the interpolation period. From Eq. (10), it can be deduced that

11 $$\begin{array}{c}{J}_{\text{cmd}}={K}_{2}\left({A}_{\text{max}}\frac{{K}_{1}\left({v}_{\text{cmd}}-{v}_{\text{act}}\right)}{\sqrt{1+{\left({K}_{1}\left({v}_{\text{cmd}}-{v}_{\text{act}}\right)\right)}^{2}}}-{a}_{act}\right)-\frac{{A}_{\text{max}}{K}_{1}{a}_{\text{act}}}{{\left(1+{K}_{1}^{2}{\left({v}_{\text{cmd}}-{v}_{\text{act}}\right)}^{2}\right)}^\frac{3}{2}}\end{array}$$

In the actual process, the jerk of the tool tip needs to satisfy the condition $|{J}_{\text{cmd}}| \le {J}_{\text{max}}$. It means Eq. (11) may not be satisfied. However, as long as ${J}_{\text{cmd}}$ doesn’t change sign, the system’s convergence direction will not change. The limit of jerk only affects convergence speed and the system remains stable.

In the A/D method, it is important to achieve the command velocity without overshoot. Near $${\left[\begin{array}{cc}{v}_{\text{cmd}}& 0\end{array}\right]}^{T}$$, as ${v}_{\text{cmd}} - {v}_{\text{act}}$ approaches 0, Eq. (1) and Eq. (11) can be approximated as

12 $$\begin{array}{c}\left\{\begin{array}{c}{a}_{\text{cmd}}\approx {A}_{\text{max}}{K}_{1}\left({v}_{\text{cmd}}-{v}_{\text{act}}\right)\\ {J}_{\text{act}}\approx \left({A}_{\text{max}}{K}_{1}\left({v}_{\text{cmd}}-{v}_{\text{act}}\right)-{a}_{\text{act}}\right)-{A}_{\text{max}}{K}_{1}{a}_{\text{act}}\end{array}\right.,When {v}_{\text{act}}\to {v}_{\text{cmd}}\end{array}$$

**Figure 3:**
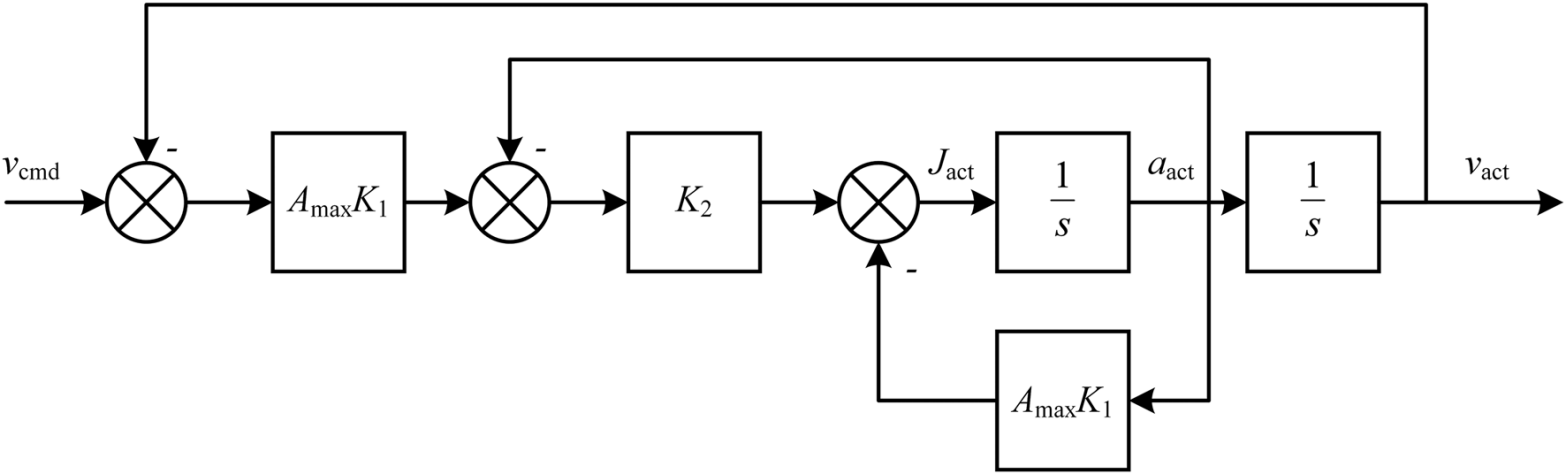
Approximated linear system.

Then the system described by Eq. (11) can be approximated as a linear system as shown in Fig. 3.

The transfer function of the system shown in Fig. 3 is

13 $$\begin{array}{c}G\left(s\right)=\frac{{A}_{\text{max}}{K}_{1}{K}_{2}}{{s}^{2}+\left({A}_{\text{max}}{K}_{1}+{K}_{2}\right)s+{A}_{\text{max}}{K}_{1}{K}_{2}}\end{array}$$

The system depicted in Eq. (13) is a typical second-order system. The natural frequency and damping ratio are

14 $$\begin{array}{c}\left\{\begin{array}{c}{\omega }_{\text{n}}=\sqrt{{A}_{\text{max}}{K}_{1}{K}_{2}}\\ \xi =\frac{{A}_{\text{max}}{K}_{1}+{K}_{2}}{2\sqrt{{A}_{\text{max}}{K}_{1}{K}_{2}}}\end{array}\right.\end{array}$$

Using Cauchy's inequality, there is

15 $$\begin{array}{c}\xi =\frac{{A}_{\text{max}}{K}_{1}+{K}_{2}}{2\sqrt{{A}_{\text{max}}{K}_{1}{K}_{2}}}\ge \frac{2\sqrt{{A}_{\text{max}}{K}_{1}}\sqrt{{K}_{2}}}{2\sqrt{{A}_{\text{max}}{K}_{1}{K}_{2}}}=1\end{array}$$

equality is achieved when ${A}_{\text{max}}{K}_{1}={K}_{2}=0$.

Therefore, the actual system's damping ratio is greater than 1, indicating that the system is overdamped and will not overshoot during convergence. It should be noted that the system described by Eq. (13) is time-continuous. Actual computer numerical control is a discrete system, which may cause some overshoot due to the discrete step size.

**Figure 4:**
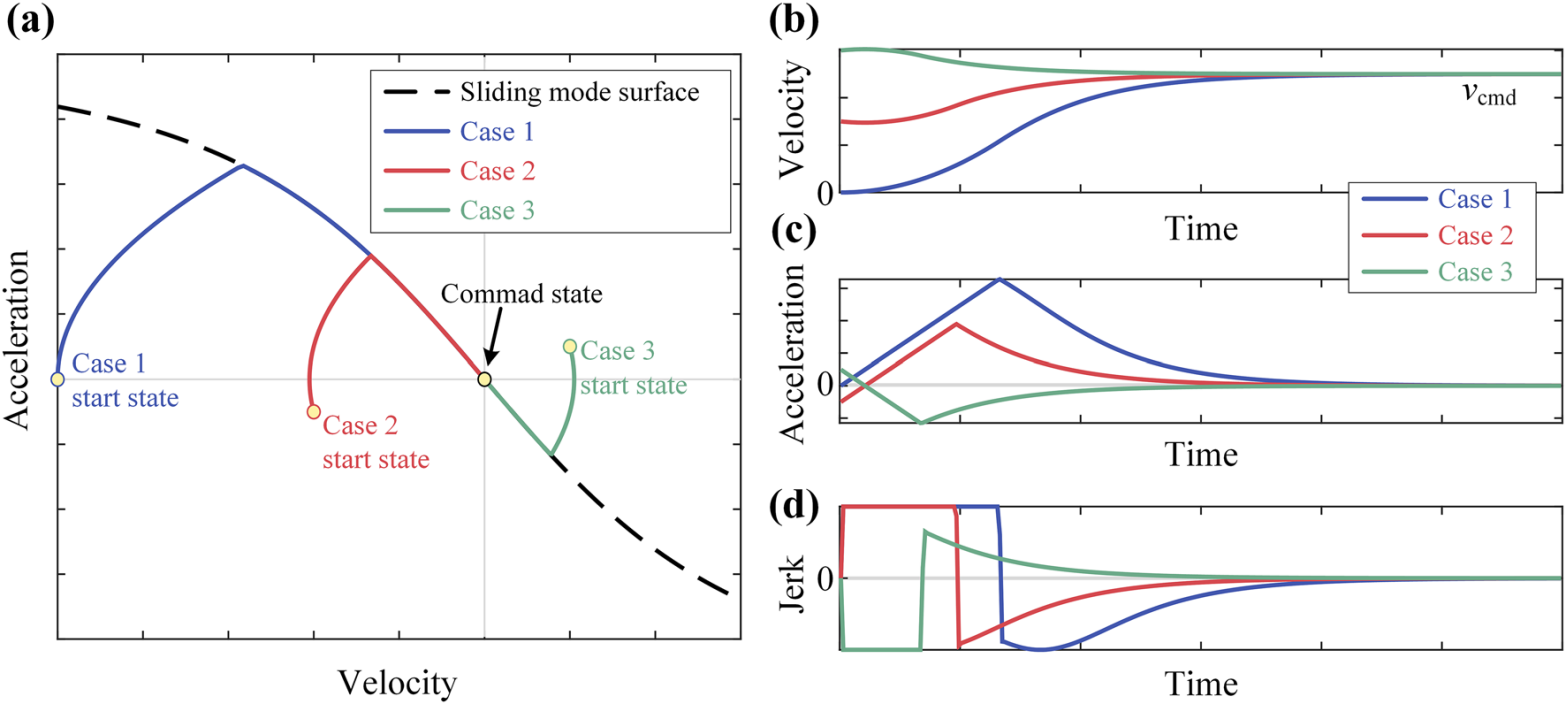
Sliding mode velocity control under 3 initial conditions: (**a**) results on phase plane;(**b**) velocity – time; (**c**) acceleration-time; (**d**) jerk-time.

Figure 4 illustrates the process of adjusting the system state to the command state using SMVC, providing three initial conditions: Case 1: Initial velocity = 0, initial acceleration = 0; Case 2: 0 < initial velocity < command velocity, initial acceleration < 0; Case 3: Initial velocity > command velocity, initial acceleration > 0. It can be observed that in all 3 cases, SMVC can effectively control system convergence to the command state.

**Figure 5:**
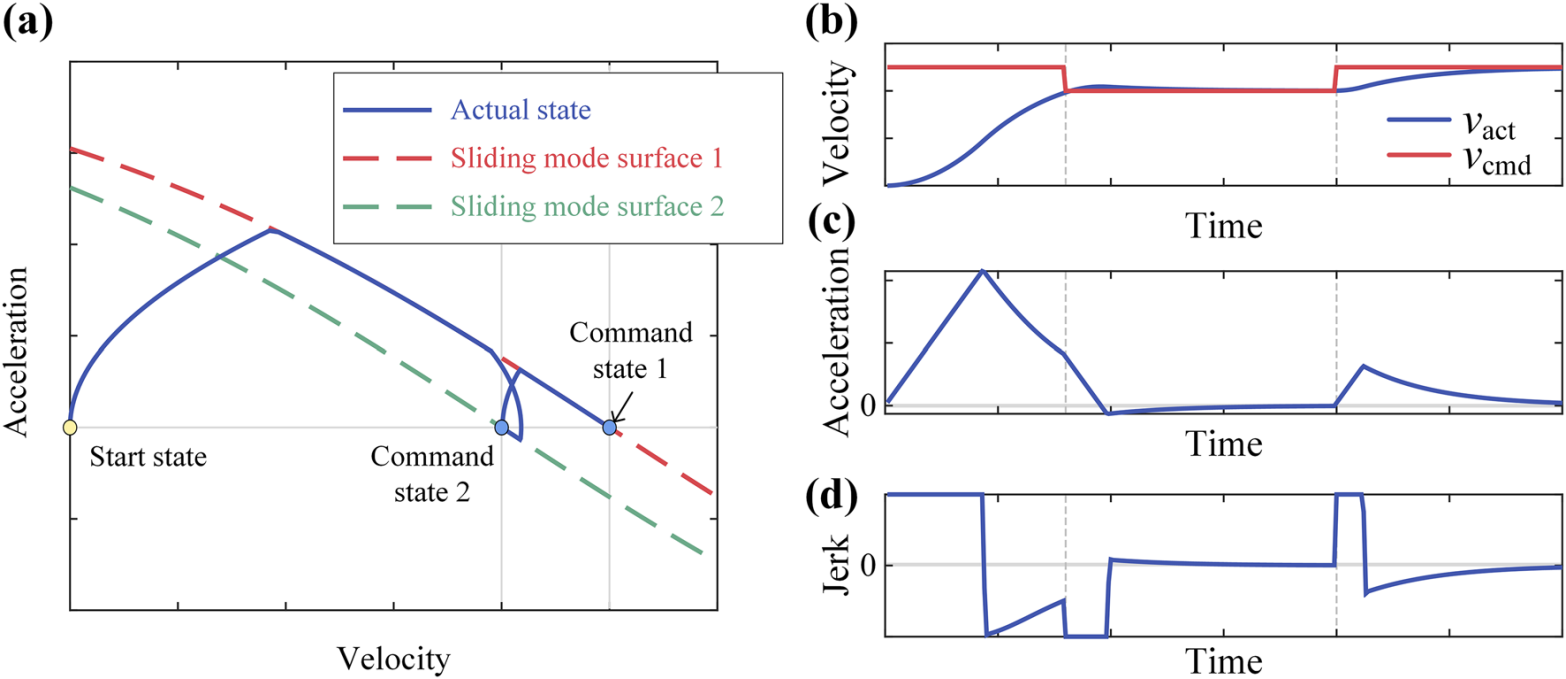
SMCVC response to override changes: (**a**) results on phase plane;(**b**) velocity – time; (**c**) acceleration-time; (**d**) jerk-time.

Figure 5 shows the response of SMVC to override changes. It shows that SMVC could flexible response the changes of override.

When the system enters the sliding mode surface, the convergence rate is determined by Eq. (1) and related to the velocity error. With the velocity error approaching 0, the convergence rate becomes very small, which can reduce the system's efficiency. Although Eq. (8) limit the value of ${K}_{1}$, for specific command velocity, the system does not pass through the jerk extrema on Eq. (1) during convergence. So, without causing overshoot, ${K}_{1}$ can be appropriately increased to improve the system's performance.

**Figure 6:**
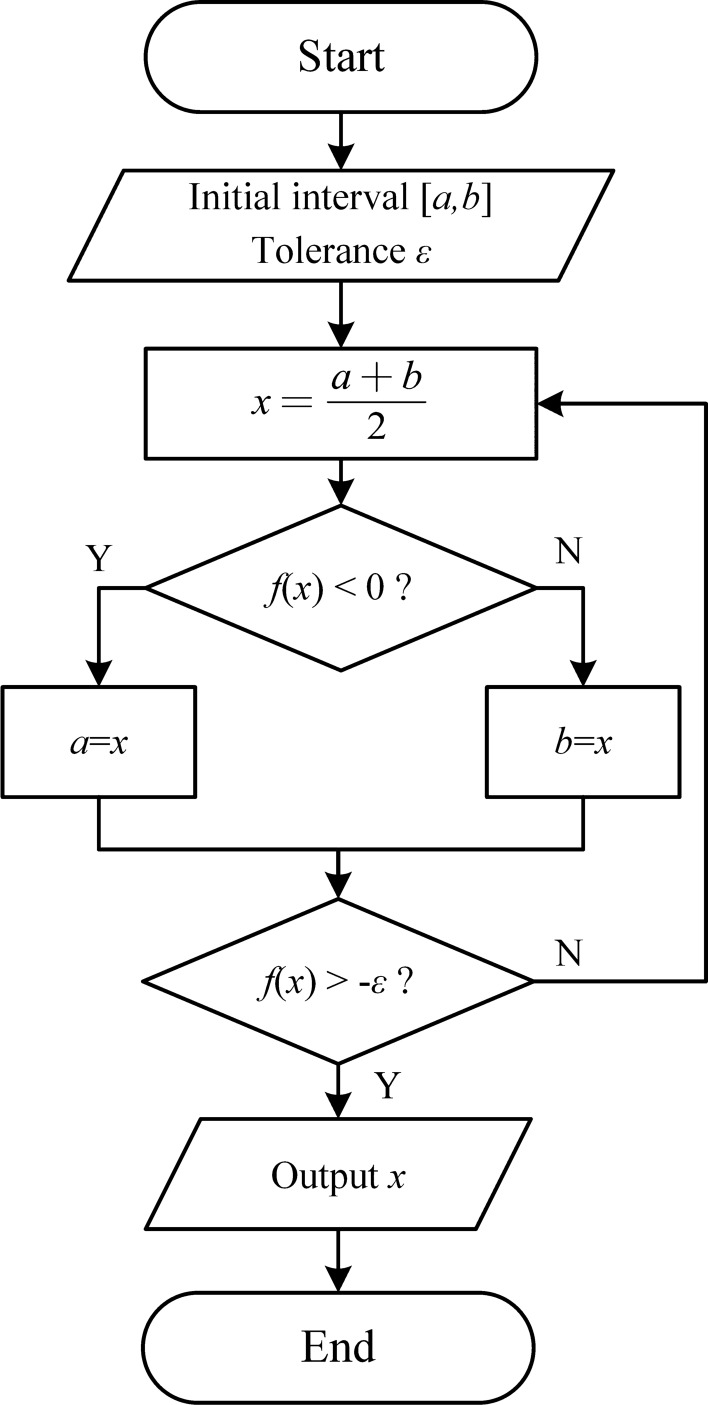
Iterative method for parameter optimization.

Due to the nonlinear characteristics of SMVC, it is difficult to obtain an analytical expression between ${K}_{1}$ and overshoot. Therefore, this paper uses an iterative method to solve for the optimal ${K}_{1}$. Let $f(x)$ be the mapping from the parameter ${K}_{1}$ to the system's overshoot. The overshoot is calculated by ${v}_{\text{max}} - {v}_{\text{cmd}}$, where ${v}_{\text{max}}$ is the maximum velocity of the system during the given time response process, and ${v}_{\text{cmd}}$ is the command velocity. This mapping can be implemented through simulation. The process of using an iterative method to find the optimal ${K}_{1}$ is shown in Fig. 6.

**Figure 7:**
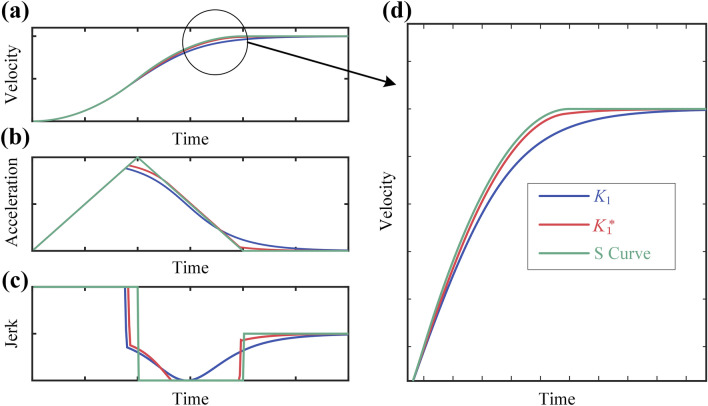
S-shaped response and SMVC response under ${K}_{1}$**,** ${K}_{1}^{*}$: (**a**) velocity–time; (**b**) acceleration-time; (**c**) jerk -time; (**d**) partial enlargement of velocity–time.

For example, with ${v}_{\text{cmd}}$ = 50 mm/s, ${A}_{\text{max}}$ = 1000 mm/s^2^, ${J}_{\text{max}}$=10,000 mm/s^3^, according to Eq. (8), ${K}_{1}$ = 0.0308 s/m is obtained. Given tolerance of 0.01 mm/s, a more optimal parameter ${K}_{1}^{*}$ = 0.0385 s/mm can be achieved. Figure 7 presents the response of SMVC under ${K}_{1}$ and ${K}_{1}^{*}$, while also comparing them to the response of the S-shaped A/D. It can be observed that the performance of the system after parameter optimization has become very close to S-shaped A/D method.

At the end of entire curve, the command state is $${\left[\begin{array}{cc}0& 0\end{array}\right]}^{T}$$.If tool system directly follows the $${\left[\begin{array}{cc}0& 0\end{array}\right]}^{T}$$, it may cause velocity to be 0 before reaching endpoint. To address this issue, this paper introduces a position-error-feedback velocity term in the endpoint command state, which changes the endpoint command state from $${\left[\begin{array}{cc}0& 0\end{array}\right]}^{T}$$ to $${\left[\begin{array}{cc}{K}_{3}\left({p}_{\text{end}}-{p}_{\text{act}}\right)& 0\end{array}\right]}^{T}$$. where ${p}_{\text{end}}$ is the endpoint position, ${p}_{\text{act}}$ is the current position, and ${K}_{3}$ is a positive coefficient. The added term ensures that the velocity of tool will not drop to 0 before reaching endpoint.

## Feedrate scheduling method

### NURBS description

The definition of an *p*-degree NURBS curve is as follows:

16 $$\begin{array}{c}C\left(u\right)=\frac{\sum_{i=0}^{n}{N}_{i,p}\left(u\right){w}_{i}{{\varvec{P}}}_{i}}{\sum_{i=0}^{n}{N}_{i,p}\left(u\right){w}_{i}}\end{array}$$

where ${N}_{i,p}\left(u\right),i=0,1,...,n$ is the basis function of the *p*-degree B-spline defined on the node sequence $${\varvec{U}}=\left[\begin{array}{cccc}{u}_{0}& {u}_{1}& \cdots & {u}_{n+p+1}\end{array}\right]$$, ${{\varvec{P}}}_{i},i=1,2,\cdots ,n$ are the control points of the NURBS curve, ${w}_{i}$ are the weight factors corresponding to the control points ${{\varvec{P}}}_{i}$, The B-spline basis function can be calculated by the de Boor-Cox recursive formulation:

17 $$\begin{array}{c}\left\{\begin{array}{c}{N}_{i,0}\left(u\right)=\left\{\begin{array}{c}0, {u}_{i}\le u\le {u}_{i+1}\\ 1, other\end{array}\right.\\ {N}_{i,p}\left(u\right)=\frac{u-{u}_{i}}{{u}_{i+p}-{u}_{i}}{N}_{i,p-1}\left(u\right)+\frac{{u}_{i+p+1}-u}{{u}_{i+p+1}-{u}_{i+1}}{N}_{i+1,p-1}\left(u\right)\end{array}\right.\end{array}$$

For five-axis machine tools, machining curve is generally described by dual NURBS curve, which is composed of 2 NURBS curve, ${\varvec{C}}(u)$ and $\left(u\right)$ , as defined by Eq. (16). And ${\varvec{C}}(u)$ represents the tool tip position and ${\varvec{O}}\left(u\right)=\frac{{\varvec{H}}\left(u\right)-{\varvec{C}}\left(u\right)}{||{\varvec{H}}\left(u\right)-{\varvec{C}}\left(u\right)||}$ represents the tool orientation vector.

The motion relationship between five physical axes and the tool tip can be expressed as

18 $$\begin{array}{c}\left\{\begin{array}{c}{q}_{s}\left(u\right)={q}_{u}\left(u\right){u}_{s}\left(u\right)\\ {q}_{ss}\left(u\right)={q}_{uu}{u}_{s}^{2}\left(u\right)+{q}_{u}\left(u\right){u}_{ss}\\ {q}_{sss}\left(u\right)={q}_{uuu}\left(u\right){u}_{s}^{3}\left(u\right)+3{q}_{uu}\left(u\right){u}_{s}\left(u\right){u}_{ss}\left(u\right)+{q}_{u}\left(u\right){u}_{sss}\left(u\right)\end{array},q=X,Y,Z\dots \right.\end{array}$$

where ${q}_{s}\left(u\right)$, ${q}_{ss}\left(u\right),{q}_{sss}\left(u\right)$ are the first order, second order and third-order derivative of the axis position with respect to the arc length $s$. ${u}_{s}\left(u\right)$, ${u}_{ss}\left(u\right),{u}_{sss}\left(u\right)$ are the first order, second order and third-order derivative of the parameter $u$ with respect to the arc length $s$.

### Chord error constraint

To ensure the discrete points can reflect the characteristics of the original curve, it is necessary to consider the constraint of the chord error.

The chord error can be expressed as:

19 $$\begin{array}{c}\delta \left(u\right)=\rho \left(u\right)-\sqrt{{\rho }^{2}\left(u\right)-{\left(\frac{{v}_{\text{t}}{T}_{\text{s}}}{2}\right)}^{2}}\le {\delta }_{\text{m}}\end{array}$$

where $\delta \left(u\right)$ is the chord error at the parameter $u$, $\rho \left(u\right)$ is the radius of curvature of the curve at $u$, ${v}_{\text{t}}$ is the feedrate, ${T}_{\text{s}}$ is the interpolation period, and ${\delta }_{\text{m}}$ is the maximum allowable chord error. The radius of curvature $\rho \left(u\right)$ can be calculated by

20 $$\begin{array}{c}\rho \left(u\right)=\frac{1}{\Vert {{\varvec{C}}}_{ss}\left(u\right)\Vert }\end{array}$$

### Servo feed axis constraint

From Eq. (18), the velocity, acceleration, and jerk of the servo feed axis $q$ can be expressed as:

21 $$\begin{array}{c}\left\{\begin{array}{c}{v}_{q}={q}_{s}\left(u\right){v}_{\text{t}}\\ {a}_{q}={q}_{ss}\left(u\right){v}_{\text{t}}^{2}+{q}_{s}\left(u\right){a}_{\text{t}}\\ {J}_{q}={q}_{sss}\left(u\right){v}_{\text{t}}^{3}+3{q}_{ss}\left(u\right){v}_{\text{t}}{a}_{\text{t}}+{q}_{s}\left(u\right){J}_{\text{t}}\end{array}\right.,q=X,Y,Z,\cdots \end{array}$$

where ${v}_{\text{t}},{a}_{\text{t}},{J}_{\text{t}}$ are the tangential velocity, tangential acceleration and tangential jerk. Each servo feed axis has a maximum allowable velocity, acceleration and jerk, so it needs to satisfy:

22 $$\begin{array}{c}\left\{\begin{array}{c}\left|{q}_{s}\left(u\right){v}_{\text{t}}\right|\le {V}_{\text{m}q}\\ \left|{q}_{ss}\left(u\right){v}_{\text{t}}^{2}+{q}_{s}\left(u\right){a}_{\text{t}}\right|\le {A}_{\text{m}q}\\ \left|{q}_{sss}\left(u\right){v}_{\text{t}}^{3}+3{q}_{ss}\left(u\right){v}_{\text{t}}{a}_{\text{t}}+{q}_{s}\left(u\right){J}_{\text{t}}\right|\le {J}_{\text{m}q}\end{array}\right.,q=X,Y,Z,\cdots \end{array}$$

### Tracking error constraint

**Figure 8:**
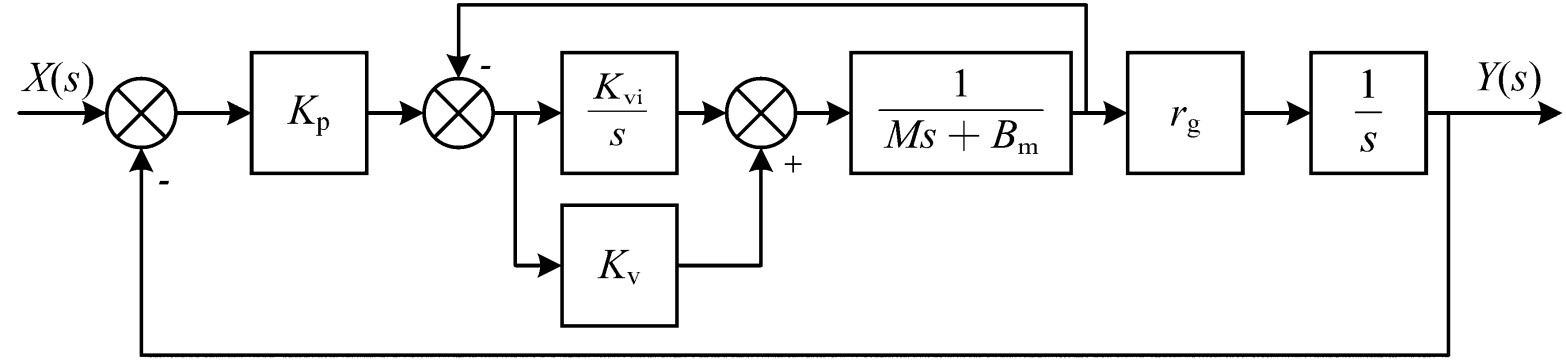
Feed axis control model.

The tracking error of tool affect the machining quality and precision^11^, and limiting the tracking error of each axis could reduce the tracking error of tool. Currently, the servo feed axis usually adopts proportional-proportional-integral control. And the control structure is shown in Fig. 8.

where ${K}_{p}$ is the proportional gain coefficient of the position loop. ${K}_{v}$ is the proportional gain coefficient of the velocity loop. ${K}_{vi}$ is the integral gain coefficient of the velocity loop. $M$ is the equivalent mass. ${B}_{m}$ is the equivalent damping coefficient and ${r}_{g}$ is the transmission coefficient.

The transfer function of the system is:

23 $$\begin{array}{c}\Phi \left(s\right)=\frac{Y\left(s\right)}{X\left(s\right)}=\frac{{K}_{\text{p}}{K}_{\text{v}}{r}_{\text{g}}s+{K}_{\text{p}}{K}_{\text{vi}}{r}_{\text{g}}}{M{s}^{3}+\left({B}_{\text{m}}+{K}_{\text{v}}{r}_{\text{g}}\right){s}^{2}+\left({K}_{\text{vi}}+{K}_{\text{p}}{K}_{\text{v}}{r}_{\text{g}}\right)s+{K}_{\text{p}}{K}_{\text{vi}}{r}_{\text{g}}}\end{array}$$

Define the tracking error $E\left(s\right)=X\left(s\right)-Y\left(s\right)$, then:

24 $$\begin{array}{c}{\Phi }_{\text{e}}\left(s\right)=\frac{E\left(s\right)}{X\left(s\right)}=\frac{M{s}^{3}+\left({B}_{\text{m}}+{K}_{\text{v}}{r}_{\text{g}}\right){s}^{2}+{K}_{\text{vi}}s}{M{s}^{3}+\left({B}_{\text{m}}+{K}_{\text{v}}{r}_{\text{g}}\right){s}^{2}+\left({K}_{\text{vi}}+{K}_{\text{p}}{K}_{\text{v}}{r}_{\text{g}}\right)s+{K}_{\text{p}}{K}_{\text{vi}}{r}_{\text{g}}}\end{array}$$

Equation (24) can be written in differential form:

25 $$ \begin{array}{*{20}c} {\frac{M}{{K_{p} K_{vi} r_{g} }}\dddot e\left( t \right) + \frac{{B_{m} + K_{v} r_{g} }}{{K_{p} K_{vi} r_{g} }}\ddot{e}\left( t \right) + \frac{{K_{vi} + K_{p} K_{v} }}{{K_{p} K_{vi} }}\dot{e}\left( t \right) + e\left( t \right) = \frac{M}{{K_{p} K_{vi} r_{g} }}J + \frac{{B_{m} + K_{v} r_{g} }}{{K_{p} K_{vi} r_{g} }}a + \frac{1}{{K_{p} K_{vi} r_{g} }}v} \\ \end{array} $$

Where $e(t),\,\dot{e}(t),\,\ddot{e}(t),\,\dddot e(t)$ is the axis tracking error and its first-order, second-order and third-order derivative, $J$ is the axis command jerk, $a$ is the axis command acceleration, and $v$ is the axis command velocity. According to the derivation of literature^26,27^, if

26 $$\begin{array}{c}\left|\frac{M}{{K}_{\text{p}}{K}_{\text{vi}}{r}_{\text{g}}}J+\frac{{B}_{\text{m}}+{K}_{\text{v}}{r}_{\text{g}}}{{K}_{\text{p}}{K}_{\text{vi}}{r}_{\text{g}}}a+\frac{1}{{K}_{\text{p}}{r}_{\text{g}}}v\right|\le E\end{array}$$

then mean $$\left|e\left(t\right)\right|\le E$$.

Therefore, the tracking error of the axis is converted into a linear combination of velocity, acceleration, and jerk of the axis. The constraint of tracking error is:

27 $$\begin{array}{c}\left|{K}_{qe3}{J}_{q}+{K}_{qe2}{a}_{q}+{K}_{qe1}{v}_{q}\right|\le {E}_{q},q=X,Y,Z,\cdots \end{array}$$

where

28 $${K}_{qe3}=\frac{{M}_{q}}{{K}_{\text{p}q}{K}_{\text{vi}q}{r}_{\text{g}q}},{K}_{qe2}=\frac{{B}_{\text{m}q}+{K}_{\text{v}q}{r}_{\text{g}q}}{{K}_{\text{p}q}{K}_{\text{vi}q}{r}_{\text{g}q}},{K}_{qe1}=\frac{1}{{K}_{\text{p}q}{r}_{\text{g}q}},q=X,Y,Z$$

### Feedrate limitation profile

The axis acceleration, jerk and tracking error are related to the actual system state. In this paper, the critical velocity satisfying above constraints can be estimated according to the state of ${a}_{t}=0,{J}_{t}=0$, and the coefficient ${K}_{\text{dyn}}$ can be used to achieve a more conservative estimate. In this paper , ${K}_{\text{dyn}}=0.9$. For satisfying the above constraints, there is:

28 $$\begin{array}{c}\left\{\begin{array}{c}{v}_{t}\le {K}_{\text{dyn}}\sqrt{\frac{{A}_{\text{m}q}}{\left|{q}_{ss}\left(u\right)\right|}},q=X,Y,Z,\dots \\ {v}_{t}\le {K}_{\text{dyn}}\sqrt[3]{\frac{{J}_{\text{m}q}}{\left|{q}_{sss}\left(u\right)\right|}},q=X,Y,Z,\dots \\ {v}_{t}\le {K}_{\text{dyn}}\frac{{E}_{q}}{{K}_{qe1}\left|{q}_{s}\left(u\right)\right|},q=X,Y,Z,\dots \end{array}\right.\end{array}$$

The feedrate limitation can be calculated as follows:

29 $$\begin{array}{c}{v}_{\text{t}}\left(u\right)\le min\left\{\frac{2\sqrt{2{\delta }_{\text{m}}\left(\rho \left(u\right)-{\delta }_{\text{m}}\right)}}{{T}_{\text{s}}},\frac{{V}_{\text{m}q}}{\left|{q}_{s}\left(u\right)\right|},{K}_{\text{dyn}}\sqrt{\frac{{A}_{\text{m}q}}{\left|{q}_{ss}\left(u\right)\right|}},{K}_{\text{dyn}}\sqrt[3]{\frac{{J}_{\text{m}q}}{\left|{q}_{sss}\left(u\right)\right|}},{K}_{\text{dyn}}\frac{{E}_{q}}{{K}_{qe1}\left|{q}_{s}\left(u\right)\right|}\right\}\end{array}$$

### Dynamic lookahead distance

In the dynamic forward-looking feedrate scheduling based on SMVC, the look-ahead distance determines whether the system can satisfy constraints and smoothly follow command. To balance efficiency and smoothness, the look-ahead distance in this paper is calculated using the braking distance, which is the shortest distance required to decelerate to 0 at the current velocity and can obtained by numerical simulation of SMVC. Due to the constraints of acceleration and jerk, the actual braking distance may be longer than in the ideal case. Then a positive coefficient ${K}_{4}$ can be multiplied to ensure sufficient look-ahead distance. Since the look-ahead distance is velocity-dependent, command velocity oscillation may occur when approaching the velocity minimum, and jitter can be suppressed using a mean filter.

### Interpolation algorithms

After obtaining the command jerk for this period, the state at the end of this interpolation period can be written:

30 $$\begin{array}{c}\left\{\begin{array}{c}{a}_{\text{act}}\left(k+1\right)={a}_{\text{act}}\left(k\right)+{J}_{\text{act}}\left(k\right){T}_{\text{s}}\\ {v}_{\text{act}}\left(k+1\right)={v}_{\text{act}}\left(k\right)+{a}_{\text{act}}\left(k\right){T}_{\text{s}}+\frac{1}{2}{J}_{\text{act}}\left(k\right){T}_{\text{s}}^{2}\\ \Delta s\left(k\right)=s\left(k+1\right)-s\left(k\right)={v}_{\text{act}}\left(k\right){T}_{\text{s}}+\frac{1}{2}{a}_{\text{act}}\left(k\right){T}_{\text{s}}^{2}+\frac{1}{6}{J}_{\text{act}}\left(k\right){T}_{\text{s}}^{3}\end{array}\right.\end{array}$$

According to paper^28^, using the second-order Taylor development, there is:

31 $$\begin{array}{c}u\left(k+1\right)=u\left(k\right)+{u}_{s}\left(u\left(k\right)\right)\Delta s\left(k\right)+\frac{1}{2}{u}_{ss}\left(u\left(k\right)\right){\left(\Delta s\left(k\right)\right)}^{2}\end{array}$$

## Simulation

To prove the effectiveness of proposed method, simulations are carried out. The simulations were carried out on a PC with Windows10 operation system. The simulation environment was Matlab 2020a and the CPU was Intel® Xeon® Silver 4110 CPU @ 2.10GHz.

The constraints and parameters for simulation of each axis are shown in Table 1:

**Table 1:** Axis constraints and parameters for simulation.

	Max velocity	Max acceleration	Max jerk	Max tracking error	*K*_*q*e1_	*K*_*q*e2_	*K*_*q*e3_
X	100 mm/s	1000 mm/s^2^	10,000 mm/s^3^	0.5 mm	0.01667 s/mm	6.1642E-5 s^2^/mm	4.5062E− 8 s^3^/mm
Y	100 mm/s	1000 mm/s^2^	10,000 mm/s^3^	0.5 mm	0.01667 s/mm	6.1642E-5 s^2^/mm	4.5062E− 8 s^3^/mm
Z	100 mm/s	1000 mm/s^2^	10,000 mm/s^3^	0.5 mm	0.01667 s/mm	6.1642E-5 s^2^/mm	4.5062E− 8 s^3^/mm
A	1 rad/s	10 rad/s^2^	100 rad/s^3^	0.1 rad	1.25E-4 s/mm	1.0836E-6 s^2^/mm	3.3796E− 10 s^3^/rad
C	0.5 rad/s	5 rad/s^2^	50 rad/s^3^	0.1 rad	1.25E-4 s/rad	1.0836E-6 s^2^/rad	3.3796E− 10 s^3^/rad

### Simulation 1: butterfly-shaped curve

**Figure 9:**
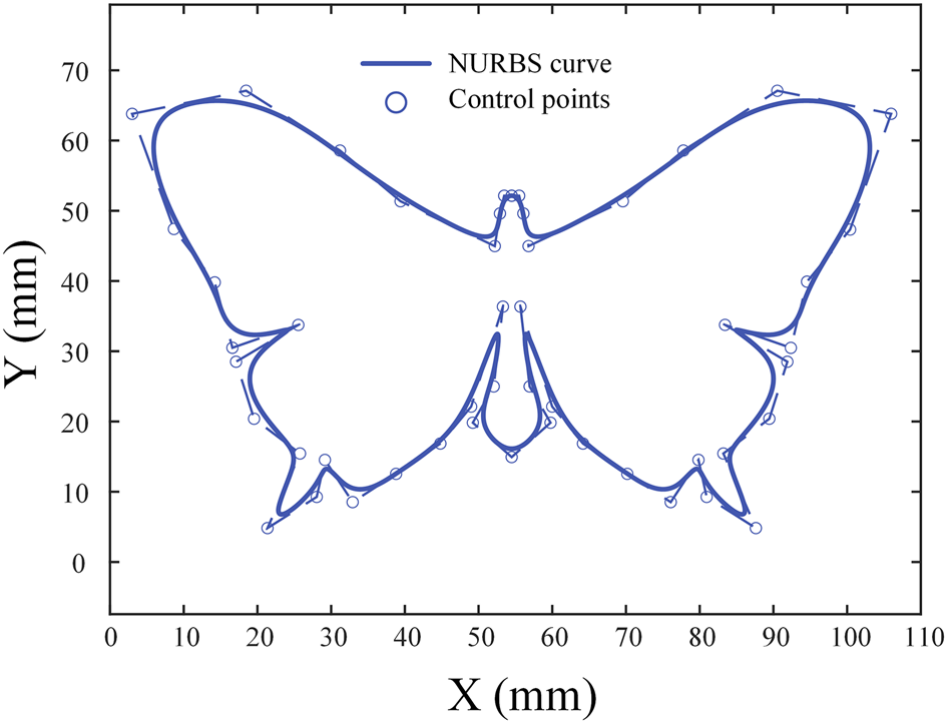
Butterfly-shaped curve.

A butterfly-shaped curve is used for simulation as shown in Fig. 9. And the curve information is shown in Appendix A Table A1.

**Figure 10:**
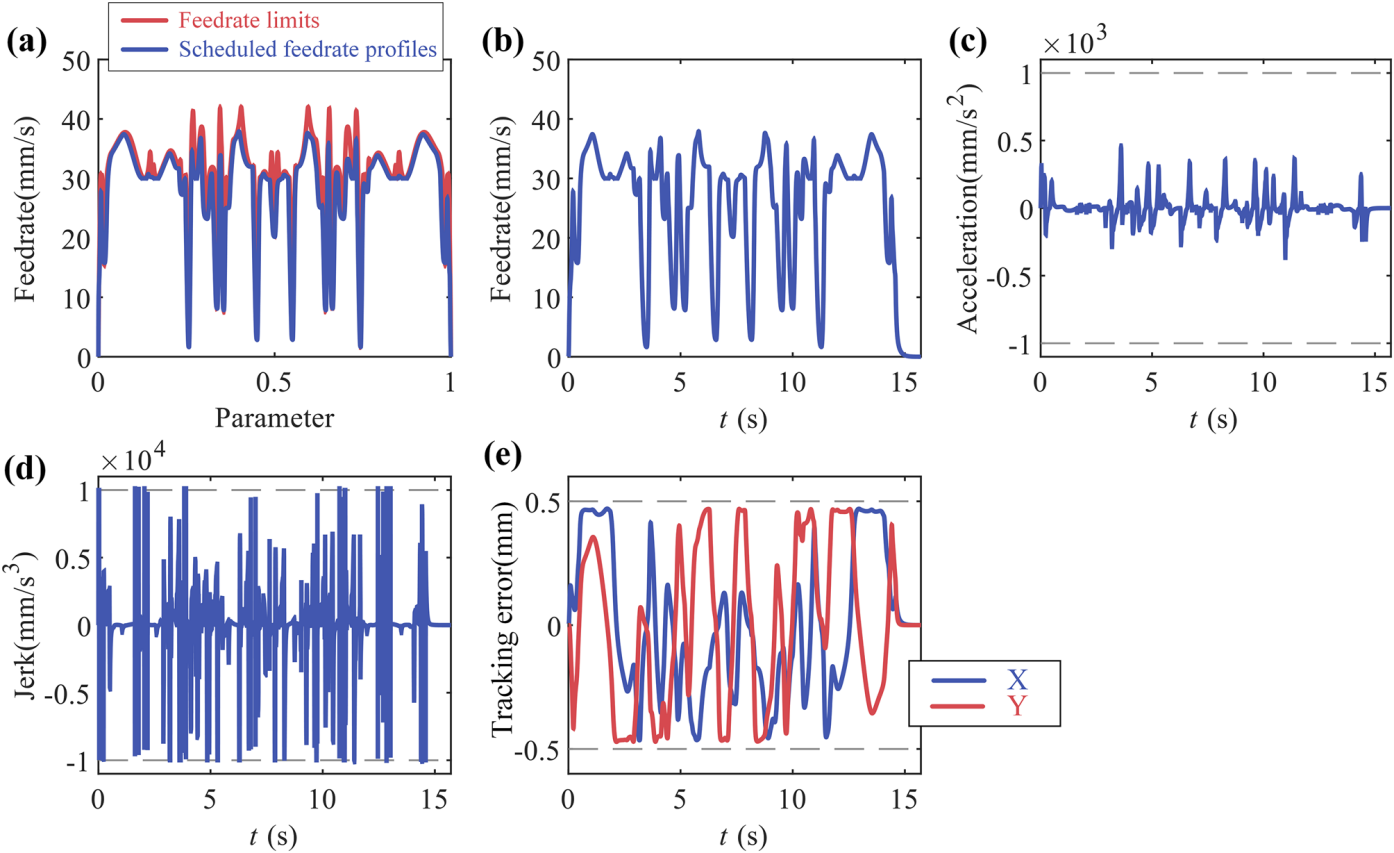
Butterfly-shaped curve simulation results: (**a**) feedrate in parameter domain; (**b**) feedrate in time domain; (**c**) tangential acceleration in time domain; (**d**) tangential jerk in time domain; (**e**) tracking error.

The tangential feedrate command is set to 50 mm/s, the maximum tangential acceleration to 1000 mm/s^2^, maximum tangential jerk to 10,000 mm/s^3^, chord error limit to 0.1μm, ${K}_{1}$ = 0.0385 , ${K}_{3}$ = 5, ${K}_{4}$ = 2. The feedrate scheduling results in parameter domain are shown in Fig. 10a and results in time domain is shown in Fig. 10b–d, the axes tracking error is shown in Fig. 10e. The results show that the method proposed could schedule feedrate satisfying the tangential velocity, acceleration and jerk constraints. And the tracking error of each axis is also within the allowable range.

**Figure 11:**
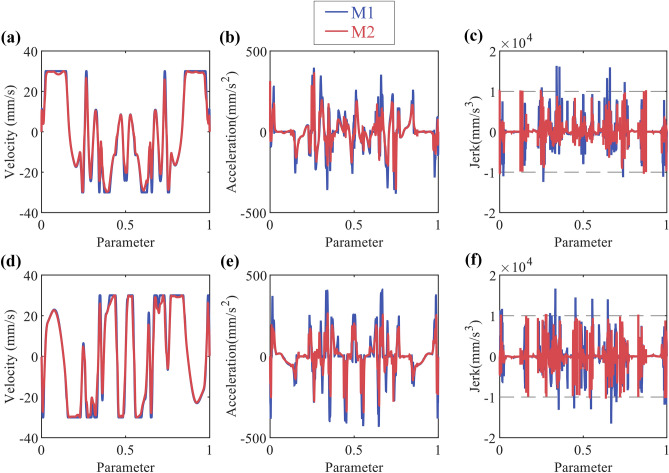
Butterfly-shaped curve simulation results: (**a**) X-axis velocity; (**b**) X-axis acceleration; (**c**) X-axis jerk; (**d**) Y-axis velocity; (**e**) Y-axis acceleration; (**f**) Y-axis jerk.

Axis acceleration and jerk are the typical dynamic constraints. Comparing the considering dynamic constraints time-optimal method^5^ (M1) with the method proposed in this paper (M2), the results shows in Fig. 11 and Table 2. The results show that the method proposed in this paper, compared to M1, effectively reduces the exceeding of dynamic constraints without significantly increasing the machining time.

**Table 2:** Performance on dynamic constraints of butterfly-shaped.

	Machine time (u ≥ 0.99999)	Acceleration exceeding constraint cycle number		Jerk exceeding constraint cycle number	Maximum exceeding constraint ratio for jerk
M1	12.908 s	0	112		X:58.5%/Y:62.07%
M2	15.362 s	0	2		X:0.49%

### Simulation 2: open-pocket curve

**Figure 12:**
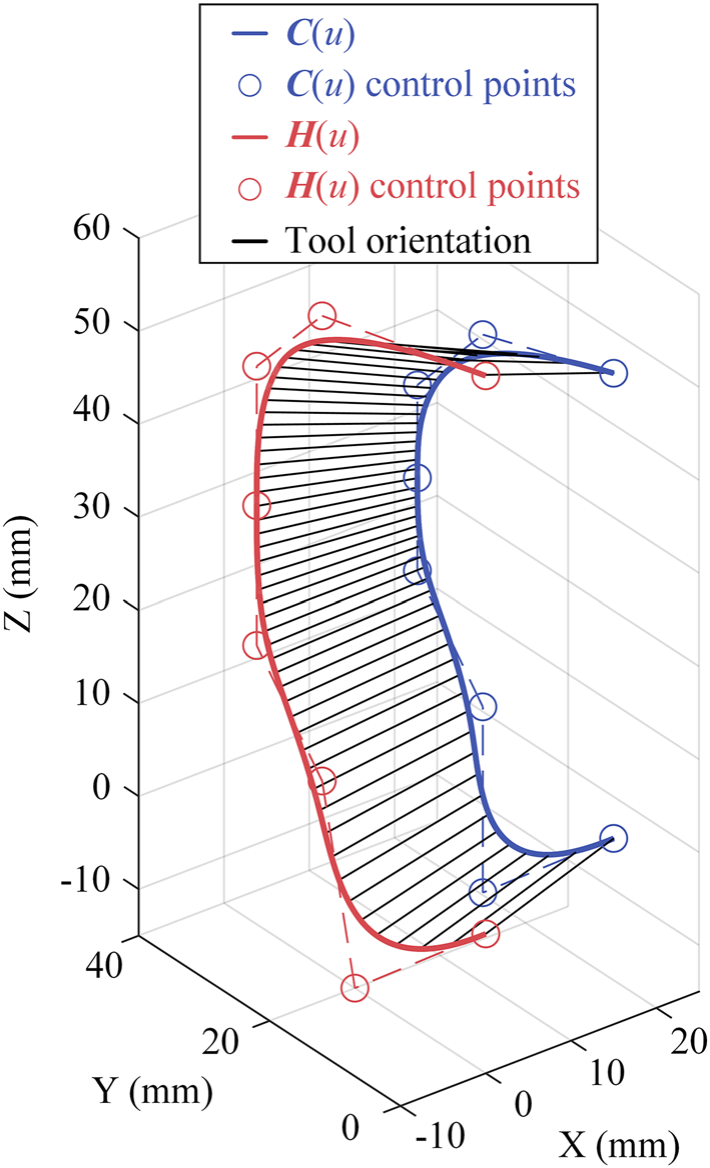
open-pocket curve.

The open-pocket curve is shown in Fig. 12. And the curve information is shown in Appendix A Table A2.

**Figure 13:**
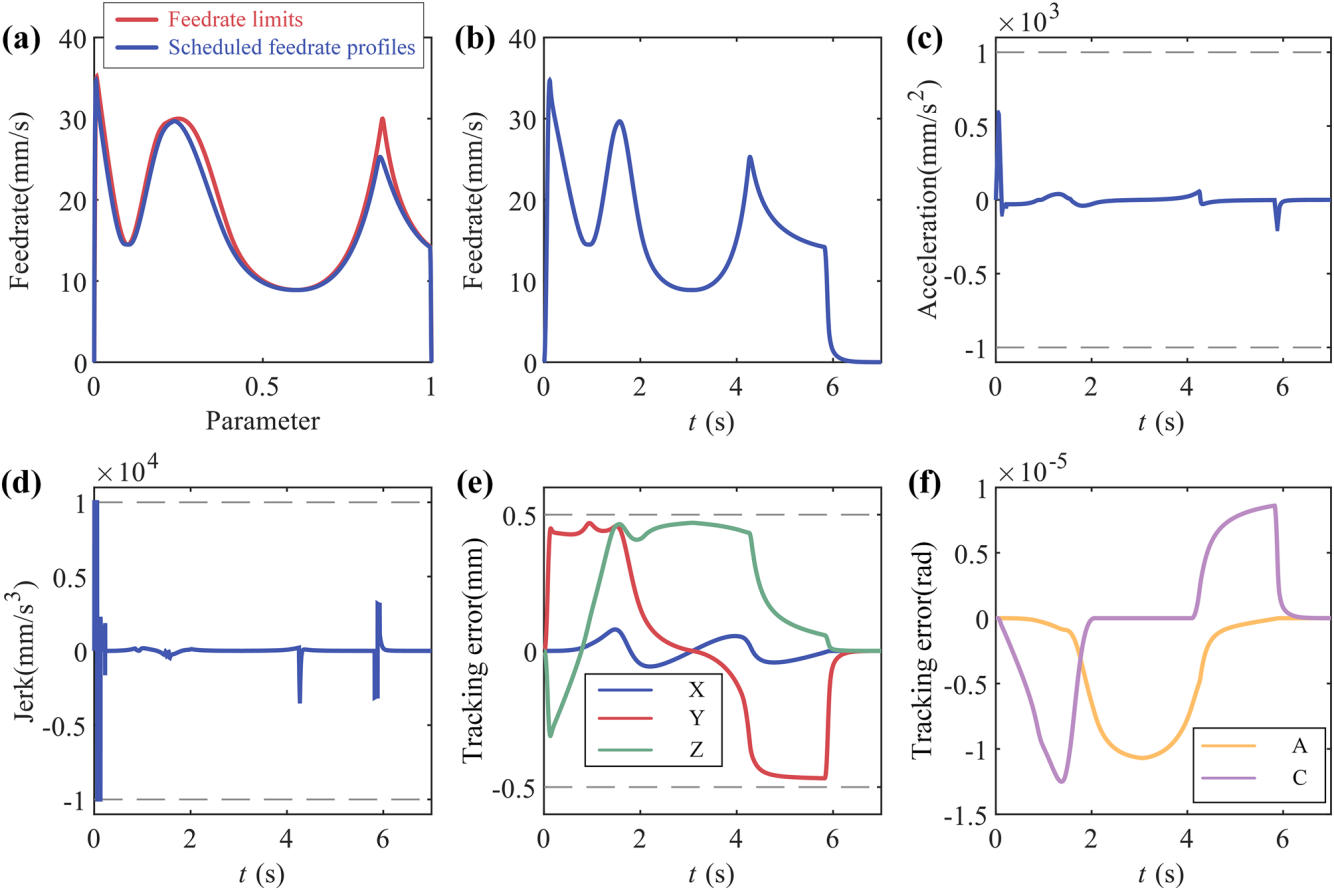
Open-pocket curve simulation results: (**a**) feedrate in parameter domain; (**b**) feedrate in time domain; (**c**) tangential acceleration in time domain; (**d**) tangential jerk in time domain; (**e**) tracking error of X,Y and Z; (**f**) tracking error of A and C.

The simulation settings are same as Simulation1. And the feedrate scheduling results in parameter domain is shown in Fig. 13a and results in time domain is shown in Fig. 13b–d, the axis tracking error is shown in Fig. 13e,f. The tangential constraints and axes tracking error are all be satisfied.

**Figure 14:**
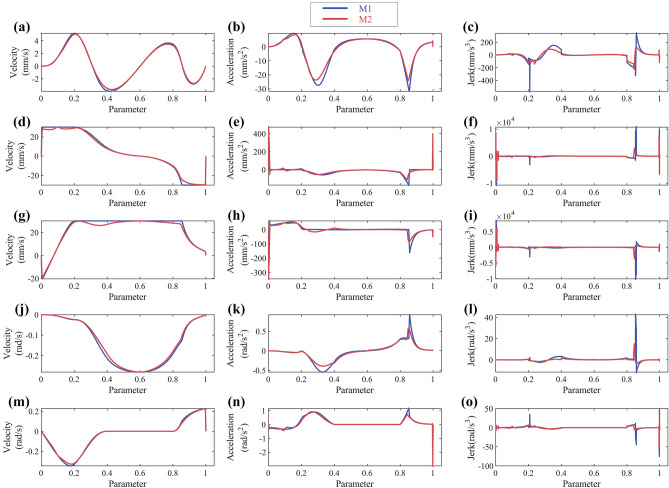
Open-pocket curve simulation results: (**a**)(**b**)(**c**) X-axis velocity/acceleration/jerk; (**d**)(**e**)(**f**) Y-axis velocity/acceleration/jerk; (**g**)(**h**)(**i**) Z-axis velocity/acceleration/jerk; (**j**)(**k**)(**l**) A-axis velocity/acceleration/jerk; (**m**)(**n**)(**o**) C-axis velocity/acceleration/jerk.

Comparing M1 with M2, the results are shown in Fig. 14 and Table 3. Although M2 method has a greater number of exceeding constraints cycles compared to M1, the exceeding constraint ratio is very minor compared to M1. Therefore, it can still be considered that the method proposed in this paper has a better performance on satisfying dynamic constraints.

**Table 3:** Performance on dynamic constrain t s of open pocket curve

	Machine time (u ≥ 0.99999)	Acceleration exceeding constraint cycle number	Jerk exceeding constraint cycle number	Maximum exceeding constraint ratio for jerk
M1	5.9540 s	0	30	Y:7.43%/Z:0.8%/C:48.5%
M2	6.6420 s	0	51	C:0.03%

## Experiment

**Figure 15:**
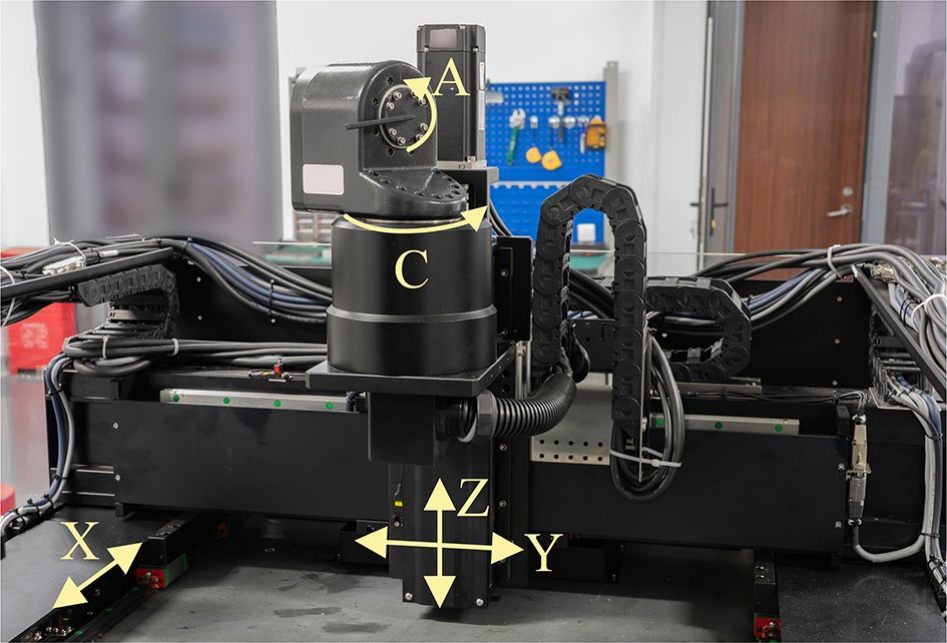
Five-axis motion platform.

To verify the real-time performance and tracking error constraints of the method proposed in this paper, experiments were carried out on a five-axis motion platform as shown in Fig. 15.

The control system of this platform is developed by TwinCAT3 and runs on a Windows 7 industrial computer an Inter® Core™ i7-6700 CPU @ 3.40 GHz. The interpolation and feedrate scheduling cycle is 2 ms. The tracking error coefficient can be identified through actual state and tracking error^4^. The identified result and constraints for each axis are shown in Table 4.

**Table 4:** Axis constraints and parameter of five-axis platform.

	Max velocity	Max acceleration	Max jerk	Max tracking error	*K*_*q*e1_	*K*_*q*e2_	*K*_*q*e3_
X	100 mm/s	1000 mm/s^2^	10,000 mm/s^3^	0.5 mm	0.01249 s/mm	1.1639E-6 s^2^/mm	3.9411E-8 s^3^/mm
Y	100 mm/s	1000 mm/s^2^	10,000 mm/s^3^	0.5 mm	0.01381 s/mm	3.5954E-5 s^2^/mm	6.0287E-7 s^3^/mm
Z	100 mm/s	1000 mm/s^2^	10,000 mm/s^3^	0.5 mm	0.01282 s/mm	4.3869E-5 s^2^/mm	2.3732E-7 s^3^/mm
A	1 rad/s	10 rad/s^2^	100 rad/s^3^	0.1 rad	0.01200 s/rad	1.6516E-5 s^2^/mm	4.4417E-8 s^3^/mm
C	0.5 rad/s	5 rad/s^2^	50 rad/s^3^	0.1 rad	0.02913 s/rad	3.1890E-4 s^2^/mm	1.6285E-7 s^3^/mm

### Experiment 1: butterfly-shaped curve with override change

The tangential feedrate command is set to 50 mm/s, maximum tangential acceleration to 1000 mm/s^2^, maximum tangential jerk to 10,000 mm/s^3^, chord error limit to 0.1μm, ${K}_{1}$ = 0.0385 , ${K}_{3}$ = 5, ${K}_{4}$ = 2. Before u ≥ 0.5, the override is set to 100%. After u ≥ 0.5, the override is adjusted to 40%, which means the tangential feedrate command is reduced to 20 mm/s. After u ≥ 0.7, the override is set back to 100%.

**Figure 16:**
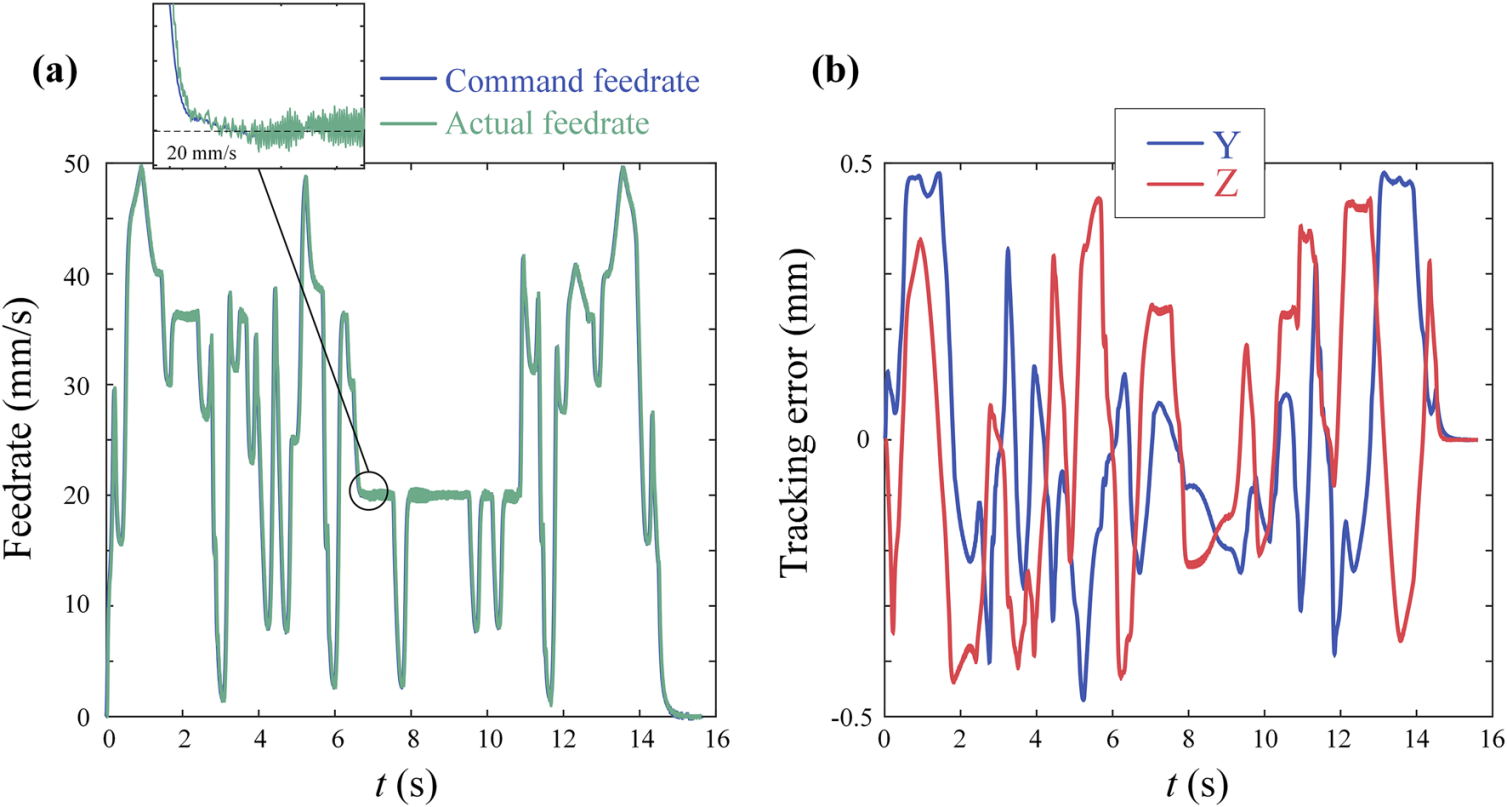
Experiment results of butter-shaped curve: (**a**) command and actual feedrate profile in time domain; (**b**) tracking error of axis.

The results are shown in Fig. 16. Figure 16a demonstrates the capability of the method proposed in this paper to adjust the override in real time. This capability provides the possibility for online control of cutting forces, which is an effective method for improving machining quality. Figure 16b shows that the maximum tracking errors for the X and Y axes are 0.4832mm and 0.4347 mm, both less than the constraint of 0.5 mm. This demonstrates the effectiveness of the tracking error constraints in this paper. and smaller tracking errors can indirectly reduce the machining contour errors, resulting in a surface with higher quality.

### Experiment 2: open-pocket curve

**Figure 17:**
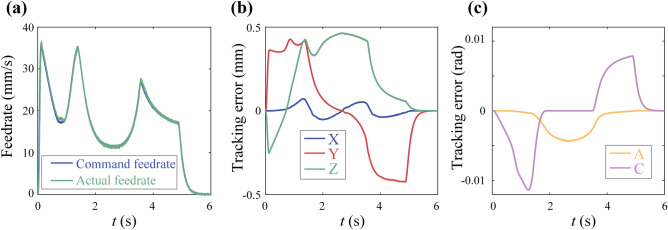
Experiment results of open-pocket curve: (**a**) command and actual feedrate profile in time domain; (**b**) tracking error of X, Y and Z; (**c**) tracking error of A and C.

Due to the dynamic performance of C axis, the experiment settings are same to experiment except ${K}_{4}$=3.2. The results are shown in Fig. 17. Figure 17a demonstrates the capability of the method proposed in this paper for 5-axis machining. Figure 17b,c indicate that the maximum tracking errors for linear axes X, Y and Z are 0.0715mm, 0.4272 mm and 0.4636 mm, all less than the constraint 0.5 mm. The maximum tracking errors of rotary axes A and C are 0.0043 rad and 0.0114 rad, both less than the constrained value of 0.1 rad.

### Computation time analysis

**Figure 18:**
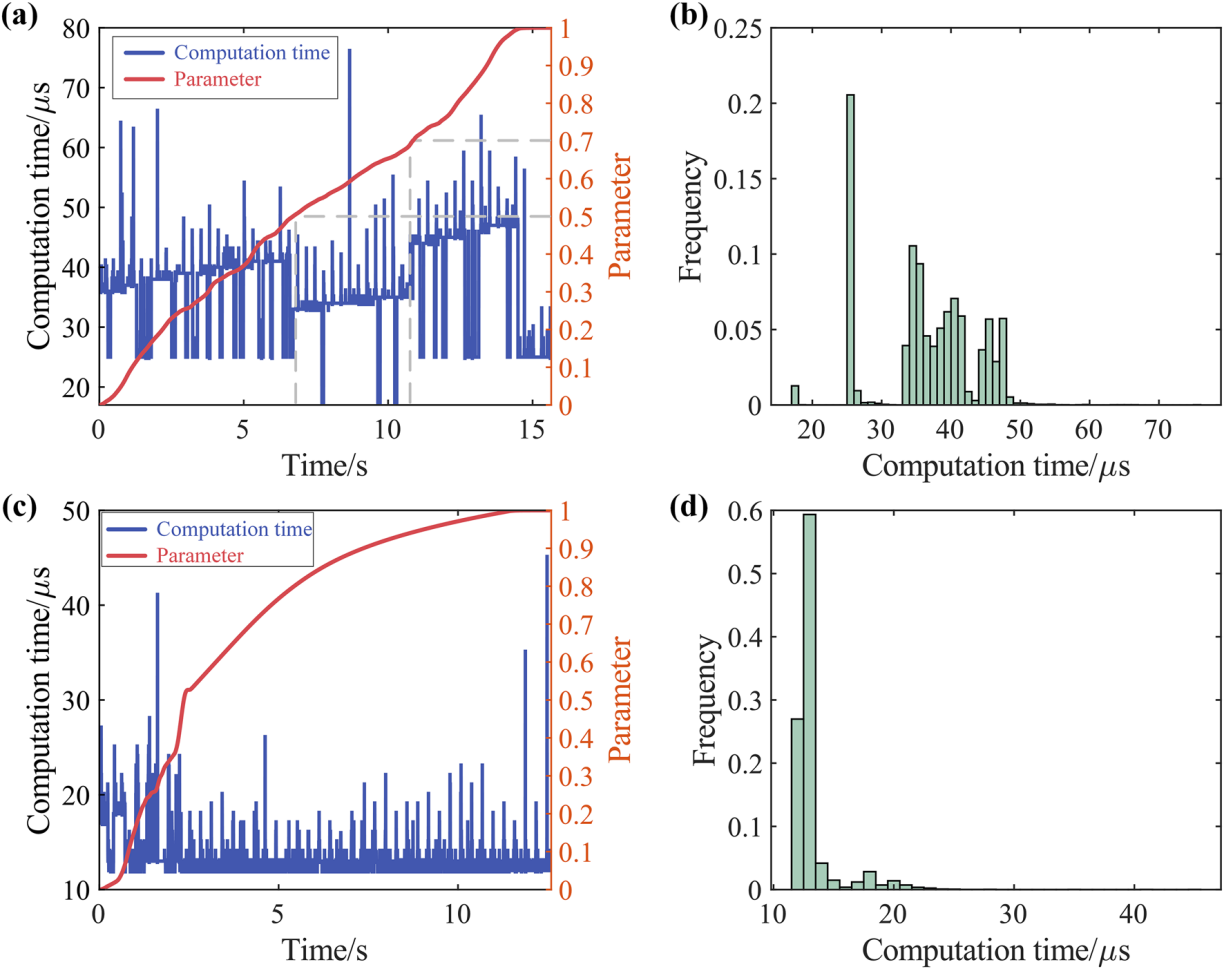
Computation time: (**a**) butterfly-shaped curve computation time and parameter timing diagram; (**b**) butterfly-shaped curve computation time histogram;(**c**) open-pocket curve computation time and parameter timing diagram; (**d**) open-pocket curve computation time histogram.

The computation time of Experiment1 and 2 is assessed by TwinCAT3 software and shown in Fig. 18. For the butterfly-shaped curve, the computation time can be kept below 50 μs in most cases, with a maximum not exceeding 80 μs. For the open-pocket curve, the computation time can be kept below 20 μs in most cases, with a maximum not exceeding 50 μs. Although it requires five-axis coordinate transformation and solution of properties of two NURBS curves, the computation time for the open-pocket curve is still significantly less than butterfly-shaped curve. This difference in computation time may be due to the differing complexities of the curves. The butterfly-shaped curve has 52 control points, while the open-pocket curve has 8 (Supplementary information).

Figure 18a illustrates the variation in computation time under different override the override was reduced when u was within [0.5,0.7]. Figure 18a shows that after the reduction of override, the average computation time decreased. A possible reason is that after feedrate has decreased, the look-ahead distance becomes shorter, which means a shorter parameter look-ahead interval and fewer iterations required to find the minimum velocity.

Figure 18 demonstrates the computational efficiency of the method proposed in this paper for both complex planar tool paths and spatial five-axis tool paths, proving the great potential of the method for real-time applications.

## Conclusion

High-precision direct interpolation of NURBS curves requires feedrate scheduling results satisfy the geometric, kinematic and dynamic constraints. Dynamic constraints are related to the current state of tool and cannot be directly constrained by velocity at sampling points. To achieve direct control of dynamic constraints, this paper proposes a dynamic look-ahead feedrate scheduling method based on sliding mode velocity control. The SMVC acceleration and deceleration method can control velocity to command velocity with any initial state and its stability has been proven. This paper also introduces the performance improvement method and end-point-reachable method. This paper analyzes the common constraints and linearizes the tracking error into a linear combination of velocity, acceleration, and jerk. The braking distance is used to estimate the look-ahead distance. The effectiveness of the proposed method in this paper is verified through simulation and experiment. In future research, selecting a smoother and more efficient sliding mode surface is a research direction The convergence speed at the endpoint also needs to be optimized, and further research is required on the selection of various coefficients.

### Supplementary Information

Supplementary Information.

## Data Availability

The datasets used or analyzed during the current study are available from the corresponding author on reasonable request.
