# Supplementary material for: Dynamic look-ahead feedrate scheduling method based on sliding mode velocity control
Source: Sci Rep. 2024 Jul 4;14:15424. doi: 10.1038/s41598-024-66203-w (PMC11224244; doi:10.1038/s41598-024-66203-w)
Supplement: Supplementary file 1 — Supplementary Information. [file 41598_2024_66203_MOESM1_ESM.docx]

**Dynamic look-ahead feedrate scheduling method based on sliding mode velocity control**

Liuquan Wang^1,2^, Qiang Liu^1,2,*^, Pengpeng Sun^3^, Shisheng Lv^1,4^, Ruijie Yang^1,5^, Zhiqi Yang^1,5^

1. School of Mechanical Engineering and Automation, Beihang University, Beijing 100191, China;

2. Jiangxi Research Institute of Beihang University, Nanchang 330096, China

3. Mechanics and Acoustics Division, National Institute of Metrology, Beijing, 100029, China

4. Beijing Engineering Technological Research Center of High-Efficient and Green CNC Machining Process and Equipment, Beijing 100191, China

5. Research and Application Center of Advanced CNC Machining Technology, State Administration of Science, Technology and Industry for National Defense, Beijing 100191, China

^*^corresponding: [qliusmea@buaa.edu.cn](mailto:qliusmea@buaa.edu.cn)

Appendix A

Table A1

Parameters of the butterfly-shape curve

| Parameters | Value |
| --- | --- |
| Degree | 3 |
| Knot vector | [0, 0, 0, 0, 0.0083, 0.015, 0.0361, 0.0855, 0.1293, 0.1509, 0.1931, 0.2273,0.2435, 0.2561, 0.2692, 0.2889,0.317, 0.3316, 0.3482, 0.3553,0.3649, 0.3837, 0.4005, 0.4269,0.451, 0.466, 0.4891, 0.5,0.5109, 0.5340, 0.5489, 0.5731,0.5994, 0.6163, 0.6351, 0.6447,0.6518, 0.6683, 0.683, 0.7111,0.7307, 0.7439, 0.7565, 0.7729,0.8069, 0.8491, 0.8707, 0.9145,0.9639, 0.985, 0.9917,1, 1, 1, 1] |
| Control points of the cutter location point | (54.493,52.139),(55.507,52.139),(56.082,49.615),(56.780,44.971),(69.575,51.358),(77.786,  58.573),(90.526,67.081),(105.973,63.801),(100.4,47.326),(94.567,39.913),(92.369,30.485),  (83.44,33.757),(91.892,28.509),(89.444,20.393),(83.218,15.446),(87.621,4.83),(80.945,9.2  67),(79.834,14.535),(76.074,8.522),(70.183,12.55),(64.171,16.865),(59.993,22.122),(55.68,  36.359),(56.925,24.995),(59.765,19.828),(54.493,14.94),(49.220,19.828),(52.06,24.994),(5  3.305,36.359),(48.992,22.122),(44.814,16.865),(38.802,12.551),(32.911,8.521),(29.152,14.  535),(28.04,9.267),(21.364,4.83),(25.768,15.447),(19.539,20.391),(17.097,28.512),(25.537,  33.75),(16.602,30.496),(14.199,39.803),(8.668,47.408),(3,63.794),(18.465,67.084),(31.197,  58.572),(39.411,51.358),(52.204,44.971),(52.904,49.614),(53.478,52.139),(54.492,52.139) |
| Weight factors | [1,1,1,1.2,1,1,1,1,1,1,1,2,1,1,5,3,1,1.1,1,1,1,1,1,1,1,1,1,1,1,1,1,1,1,1.1,1,3,5,1,1,2,1,1,1,1,1,1,1,1.2,1,1,1] |

Table A2

Parameters of the open-pocket curve

| Parameters | Value |
| --- | --- |
| Degree | 3 |
| Knot vector | [0, 0, 0, 0, 0.2, 0.4, 0.6, 0.8, 1, 1, 1, 1] |
| Control points of the cutter location point | (5, 0, 0), (-10, 20, 0), (10, 20, 0), (20, 30, 0), (30, 30,0), (40, 30, 0),(50, 20, 0), (55, 0, 0) |
| Control points of the cutter axis point | (0, 0, 15), (-15, 20, 15), (5, 25, 15), (15, 35, 15), (30,35, 15), (45, 35, 15), (55, 25, 15), (60, 0, 15) |
| Weight factors | [1, 1, 1, 1, 1, 1, 1, 1] |
